# Alternative graphical displays for the monitoring of epidemic outbreaks, with application to COVID-19 mortality

**DOI:** 10.1186/s12874-020-01122-8

**Published:** 2020-10-06

**Authors:** Thomas Perneger, Antoine Kevorkian, Thierry Grenet, Hubert Gallée, Angèle Gayet-Ageron

**Affiliations:** 1 Division of clinical epidemiology, Geneva University Hospitals, and Faculty of medicine, University of Geneva, Geneva, Switzerland; 2 Teem Photonics, 61 Chemin du Vieux Chêne, 38240 Meylan, France; 3 Neel Institute, Université Grenoble Alpes, Grenoble, France; 4 Institute of Environmental Geosciences, Université Grenoble Alpes, Grenoble, France

**Keywords:** Epidemic curve, Growth rate, COVID-19

## Abstract

**Background:**

Classic epidemic curves – counts of daily events or cumulative events over time –emphasise temporal changes in the growth or size of epidemic outbreaks. Like any graph, these curves have limitations: they are impractical for comparisons of large and small outbreaks or of asynchronous outbreaks, and they do not display the relative growth rate of the epidemic. Our aim was to propose two additional graphical displays for the monitoring of epidemic outbreaks that overcome these limitations.

**Methods:**

The first graph shows the growth of the epidemic as a function of its size; specifically, the logarithm of new cases on a given day, N(t), is plotted against the logarithm of cumulative cases C(t). Logarithm transformations facilitate comparisons of outbreaks of different sizes, and the lack of a time scale overcomes the need to establish a starting time for each outbreak. Notably, on this graph, exponential growth corresponds to a straight line with a slope equal to one. The second graph represents the logarithm of the relative rate of growth of the epidemic over time; specifically, log_10_(N(t)/C(t-1)) is plotted against time (t) since the 25th event. We applied these methods to daily death counts attributed to COVID-19 in selected countries, reported up to June 5, 2020.

**Results:**

In most countries, the log(N) over log(C) plots showed initially a near-linear increase in COVID-19 deaths, followed by a sharp downturn. They enabled comparisons of small and large outbreaks (e.g., Switzerland vs UK), and identified outbreaks that were still growing at near-exponential rates (e.g., Brazil or India). The plots of log_10_(N(t)/C(t-1)) over time showed a near-linear decrease (on a log scale) of the relative growth rate of most COVID-19 epidemics, and identified countries in which this decrease failed to set in in the early weeks (e.g., USA) or abated late in the outbreak (e.g., Portugal or Russia).

**Conclusions:**

The plot of log(N) over log(C) displays simultaneously the growth and size of an epidemic, and allows easy identification of exponential growth. The plot of the logarithm of the relative growth rate over time highlights an essential parameter of epidemic outbreaks.

## Background

During the COVID-19 pandemic of 2020, epidemic curves have become commonplace in scientific and mainstream media [1, 2]. Most curves display the daily number of events over time or the cumulated number of events over time. These epidemic curves are effective for communicating the size (cumulative number of cases) and absolute growth rate (new cases per day) of the outbreak. However, no single graphical method can effectively convey all relevant aspects of an epidemic. Classic epidemic curves (displayed in natural units) have the following shortcomings:

- New cases and cumulative cases, both of which are of interest, are not shown jointly using the same metric. Cumulative cases correspond to the area under the curve of new cases over time, and the daily accrual of cases corresponds to the slope of the cumulative cases over time, but evaluating these quantities by visual inspection is difficult.
- Numbers involved in epidemics can span several orders of magnitude over time or when countries of different sizes and epidemic states are compared. Classic linear graphs do not allow the simultaneous visualization of the different scales involved, and using several graphs to emphasize different parts obscures the comparison of absolute numbers.
- Asynchronous outbreaks may be difficult to compare, as the definition of t = 0 relies inevitably on sparse data, and depends on efforts expended to identify the earliest cases of the disease.
- The relative rate of growth (new cases divided by the cumulated total to date) is an important metric: it is constant when the epidemic growth is exponential, and typically it decreases over time as the epidemic progresses. However it cannot be read directly from classic epidemic curves.
- Day-to-day variance in event counts makes it difficult to ascertain when the peak of the epidemic has occurred, and how fast the slow-down of the outbreak is progressing.

In searching for alternative graphical representations, we sought to apply the following principles:

- Use of logarithm transformations to allow the simultaneous visualization of different orders of magnitude in the data, including direct comparisons of small and large outbreaks.
- Simultaneous display of two key characteristics of epidemic outbreaks: new daily cases (growth) and cumulative cases (size).
- Use of a representation in which canonical cases (exponential growth, sub-exponential growth, linear growth) appear as easily identified visual patterns (straight line with unit slope, line with slope < 1, flat line).
- Choice of axes to display relative growth over time

We propose here two graphs that apply these principles.

## Methods

We propose two graphs that are easily obtained from daily counts of events.

- Logarithm of new daily events as a function of the logarithm of cumulative events

The logarithm of the daily increase in events (new cases or deaths, N) is plotted against the logarithm of the cumulative count of events (total cases or deaths, C), so that both the rate of progression of the epidemic and its total size are jointly readable.

Furthermore, this plot allows easy detection of exponential, sub-exponential, and linear growth. Under exponential growth, the cumulated number of events at time t is defined as C(t) = b_0_∙(1 + r)^t^, where b_0_ is the seed number of cases at t = 0, and r is the relative increase over one unit of time, typically 1 day. Note that a constant value of r defines exponential growth. This process implies that C(t) equals C(t – 1)∙(1 + r). The number of new daily cases N(t) is C(t) – C(t – 1), thus N(t) = C(t – 1)∙r, or N(t) = C(t)∙r/(1 + r). Taking logarithms of this equation yields:

$$ \log \left(\mathrm{N}\left(\mathrm{t}\right)\right)=\log \left(\mathrm{C}\left(\mathrm{t}\right)\right)+\log \left(\mathrm{r}\right)-\log \left(1+\mathrm{r}\right) $$

Thus when growth of C(t) is exponential the plot of log(N(t)) over log(C(t)) is a straight line with slope equal to + 1.

When the slope of log(N(t)) over log(C(t)) differs from 1, the growth is not exponential; the relative growth parameter r must vary over time, and is denoted by r(t). From N(t) = C(t – 1)∙r(t) and C(t – 1) = C(t) – N(t), which are true regardless of the epidemic growth process, it follows that:

$$ \log \left(\mathrm{N}\left(\mathrm{t}\right)\right)=\log \left(\mathrm{C}\left(\mathrm{t}\right)\right)+\log \left(\mathrm{r}\left(\mathrm{t}\right)\right)-\log \left(1+\mathrm{r}\left(\mathrm{t}\right)\right) $$

In this general case the plot of log(N(t)) over log(C(t)) is not constrained to be a straight line with unit slope. However straight lines, when applicable, represent distinctive growth patterns of epidemics, i.e., the time-dependence of C(t) [3]. A straight line with slope s indicates a power law relation of the type N(t) = k C(t)^s^; this implies that each time C(t) increases two-fold or ten-fold, N(t) is multiplied by 2^s^ or 10^s^. The special case s = 0 thus corresponds to a constant value of N(t), hence a linear growth of C(t). A value of s such that 0 < s < 1 corresponds to sub-exponential growth. When s equals 1, the growth becomes exponential. Finally if s > 1, the growth is supra-exponential.

b) Logarithm of relative growth rate over time

Most epidemics exhibit exponential or near-exponential growth only at their very beginning, then slow down as the number of susceptible individuals shrinks. When growth is exponential, the day-to-day multiplicative factor (1 + r) is constant, and so is r. But r can and does change over time. The time-varying relative growth rate r(t) can be estimated by the ratio of new cases N(t) divided by the cumulative total of the previous day C(t-1), since N(t) = C(t – 1)∙r(t). In the course of the epidemic the relative growth rate decreases and eventually reaches 0 as the epidemic ends. How fast this decrease occurs is therefore of primary public health interest. Because r has a lower bound of 0 and may decay exponentially over time (such that r(t) = r_0_∙exp.(−λ∙t)), we propose to plot the logarithm of the relative growth rate (log(r)) versus time. On this plot a negative slope would represent -λ, i.e., the rate of decay of the relative growth rate (on a log scale).

### Applications

We used two applications to illustrate the use of these graphs: the classic Susceptible-Infected-Removed (SIR) model [4], and multi-country comparisons of the daily numbers of deaths attributed to COVID-19.

- SIR model

The SIR model is defined by equations that describe the transitions between the three states, S, I, and R (furthermore, P is the total population, *P* = S + I + R, β is the infection rate per unit time, and γ is the removal rate per unit time):

$$ \mathrm{dS}/\mathrm{dt}=-\upbeta \mathrm{SI}/\mathrm{P} $$

$$ \mathrm{dI}/\mathrm{dt}=\upbeta \mathrm{SI}/\mathrm{P}-\upgamma \mathrm{I} $$

$$ \mathrm{dR}/\mathrm{dt}=\upgamma \mathrm{I} $$

Deaths are a fixed proportion of the removed. We simulated an arbitrary epidemic using β = 0.4, γ = 0.04, and probability of death among the removed of 0.4, and displayed the number of deaths using three methods: a) new deaths N(t) over time, the classic epidemic curve, and the two proposed graphs, b) log(N(t)) as a function of log(C(t)), and c) log(N(t)/C(t – 1)) =log( r(t)) over time. Note that changing the model parameters changes the spread of death occurrences over time, but the general shape remains unchanged.

b) Deaths attributed to COVID-19

We applied the proposed methods to daily counts of deaths attributed to COVID-19 reported by the European Centre for Disease Prevention and Control in selected countries [5]. The data are publicly available. We selected 11 countries from Europe and 11 from outside Europe. We retained countries that experienced > 1000 deaths attributed to COVID-19, and that displayed distinctive epidemic patterns, for illustration purposes (we did not aim to provide a comprehensive world-wide picture of the pandemic). We used deaths and not cases because we presumed death counts to be more reliable. We used all deaths reported between the first death attributed to COVID-19 in the country and June 5, 2020, with 2 exceptions: death counts from China were stopped on April 16, 2020, before the addition of 1290 deaths that were identified retrospectively, and death counts from Spain were stopped on May 24, 2020, before an adjustment that subtracted 1918 deaths from the total. For the plot of the relative growth rate over time, we defined time zero as the day of the 25th death in each country, and we used logarithms in base 10, to facilitate the numerical interpretation of r(t).

To smooth trends we applied non-parametric regression [6]. The analysis and graphs were performed using IBM SPSS Statistics version 25.

## Results

- SIR model

**Fig. 1:**
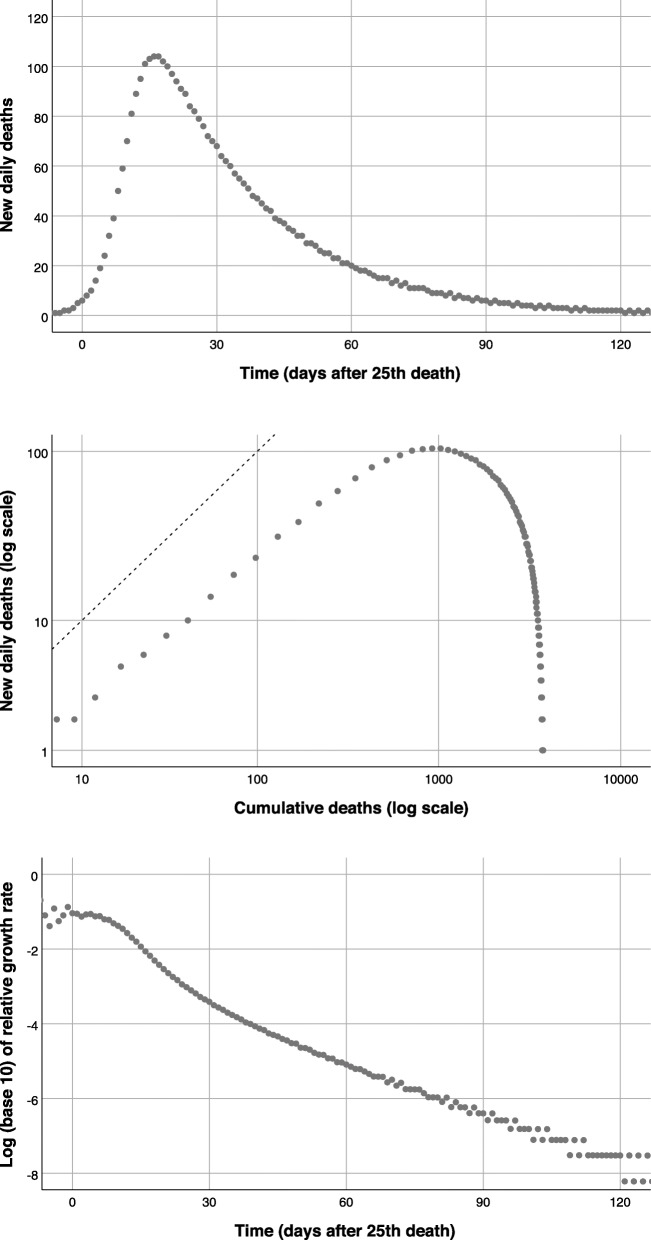
Representations of the accrual of deaths from the SIR model: daily deaths over time (upper panel), log of daily deaths versus log of total deaths (middle panel; the dotted line shows the slope expected with exponential growth), and log of relative growth rate over time (lower panel)

The number of deaths per day followed a familiar pattern: events increase, reach a peak, then decrease progressively (Fig. 1upper panel). The plot of log(N) versus log(C) showed an initial straight line, followed by a progressive downturn (Fig. 1middle panel). The plot of log(r) over time exhibited a regular downward trend, interrupted by a flatter section in the initial phase of the epidemic (Fig. 1lower panel), representing near-exponential growth.

b) Deaths attributed to COVID-19

**Fig. 2:**
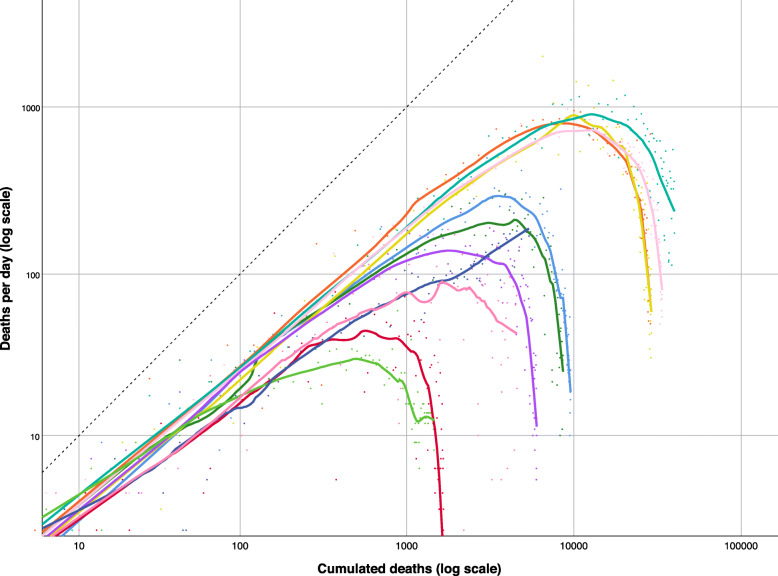
Logarithm of daily number of deaths attributed to COVID-19 versus logarithm of cumulative number of deaths in 11 European countries, as of June 5, 2020 (from top right down): United Kingdom (teal), Italy (light pink), France (mustard), Spain (orange), Belgium (light blue), Germany (dark green), Russia (navy blue), Netherlands (purple), Sweden (dark pink), Switzerland (red), Portugal (light green). Smoothed lines were obtained by non-parametric regression. Dotted line is the identity function, parallel to exponential growth

**Fig. 3:**
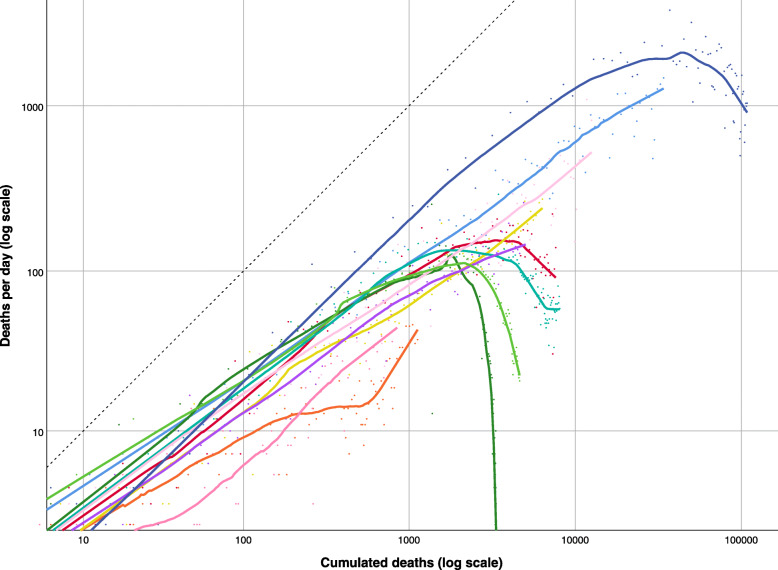
Logarithm of daily number of deaths attributed to COVID-19 versus logarithm of cumulative number of deaths in 11 non-European countries, as of June 5, 2020 (from top right down): USA (navy blue), Brazil (light blue), Mexico (light pink), India (mustard), Canada (red), Iran (teal), Peru (purple), Turkey (light green), China (dark green), Egypt (orange), South Africa (dark pink). Smoothed lines were obtained by non-parametric regression. Dotted line is the identity function, parallel to exponential growth

The plot of the logarithm of deaths versus logarithm of cumulative deaths in European countries (Fig. 2) showed in most cases a pattern similar to that observed under the SIR model: the initial growth was sustained, typically not quite exponential (most slopes appear less steep than 1), then the curve broke and trended downward. The downturn was only partial in Sweden (dark pink), and had not yet happened in Russia (navy blue). In Portugal (light green), a flat section occured before the daily counts have descended into single digits, suggesting a low-grade persistence of disease activity. In non-European countries (Fig. 3) the downturn was completed in China (dark green), where daily death counts fell into single digits, and was well on its way in Turkey (light green). Daily counts from Iran (teal) displayed a flat section that interrupted the downturn, similarly to Portugal. The downturn was still ongoing in the USA (navy blue) and in Canada (red). Epidemics of COVID-19 deaths were still undergoing sub-exponential growth in Brazil (light blue), Mexico (pink), Peru (purple), India (mustard), Egypt (orange), and South Africa (dark pink).

c) Relative growth rates of COVID-19 epidemics

**Fig. 4:**
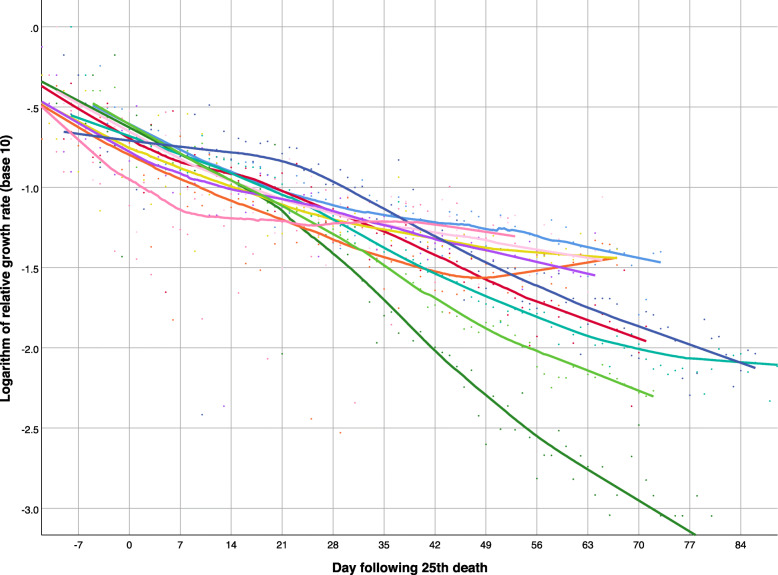
Logarithm of relative growth rate of deaths attributed to COVID-19 over time in 11 European countries, as of June 5, 2020 (from top right down): Russia (navy blue), Italy (light pink), Sweden (dark pink), Portugal (light green), United Kingdom (teal), France (mustard), Spain (orange), Germany (dark green), Netherlands (purple), Belgium (light blue), Switzerland (red). Smoothed lines were obtained by non-parametric regression

**Fig. 5:**
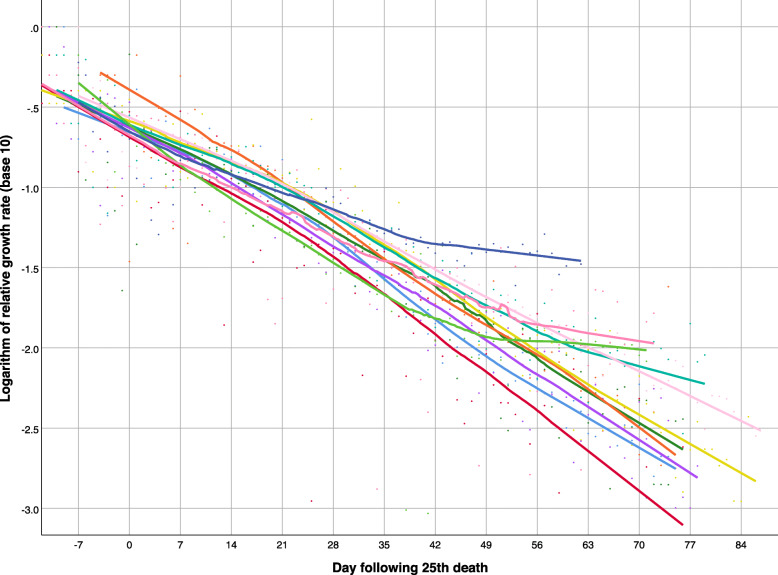
Logarithm of relative growth rate of deaths attributed to COVID-19 over time in 11 non-European countries, as of June 5, 2020 (from top right down): Brazil (light blue), South Africa (dark pink), Mexico (light pink), India (mustard), Peru (purple), Egypt (orange), USA (navy blue), Canada (red), Turkey (light green), Iran (teal), China (dark green). Smoothed lines were obtained by non-parametric regression

In most European countries (Fig. 4), the negative trend in the logarithm of the relative growth rate was nearly linear, but the decrease was less pronounced in Russia (navy blue), Portugal (light green), Sweden (dark pink), and the United Kingdom (teal) in recent weeks. More variability is seen among non-European countries (Fig. 5): the decrease in the relative growth rate was steepest in China (dark green), and persistently decreasing in Turkey (light green), Iran (teal) - with the exception of the last few weeks, Canada (red), and the USA (navy blue), but the growth rates have remained nearly flat in Brazil (light blue), Mexico (light pink), India (mustard), Peru (purple), South Africa (dark pink), and Egypt (orange).

## Discussion

We propose two graphical displays of epidemics that may be helpful for the monitoring of epidemic outbreaks. The plot of the log of new cases versus the log of cumulative cases provides a simultaneous assessment of growth and size on equivalent and easily read scales. The logarithm transformation facilitates comparisons of large and small outbreaks; it demonstrates the fundamental similarity of the epidemic process independent of population size, without scale-related distortions that affect classic epidemic curves. While this method is not specific to any particular disease, it was described independently by several teams in the context of the COVID-19 pandemic, including the authors of this paper, and the authors of the site Covid Trends [3, 7].

Applied to COVID-19 deaths in selected countries, these plots showed a common pattern (the appearance of a crashing wave) that is partly a consequence of the rescaling due to the logarithm transformations, but that also highlights a phenomenon – the peak of the epidemic – that may be difficult to discern from event counts over time. These plots allow direct comparisons of smaller and larger outbreaks, and identify at a glance outbreaks that have not yet reached their peak. This may be particularly useful to convey a synthetic view, e.g., for public communication.

An unusual feature is that the plot of log(N) over log(C) does not explicitly display calendar time, although since C(t) increases with time, observations are ranked temporally along the abscissa. This can be an advantage when various epidemic outbreaks start at different times; indeed, defining a precise date for t = 0 may be challenging when initial cases occur sporadically. But the lack of an explicit time scale precludes comparisons of the timing of outbreaks. Temporal progression can be represented by dynamic graphs that show an animation of the epidemic curves over time [7, 8].

Another property of the plot of log(N) over log(C) is the lack of a population denominator: only event counts are used, regardless of the size of the underlying population at risk. However, epidemic outbreaks rarely affect a whole country uniformly, so the relevant denominator (the population potentially exposed to the infectious agent) is difficult to define.

The plot of the logarithm of the relative growth rate over time displays the evolution of a key attribute of any epidemic, one that is difficult to ascertain from classic epidemic curves. Application to COVID-19 deaths shows that the relative growth rate was decreasing from the beginning in most countries, even as daily death counts were still on the rise. A notable exception was the USA which first displayed a plateau associated to an almost pure exponential growth. The plot also shows that despite the general downward trend over time, some countries experienced considerably higher relative growth rates than others.

### Limitations

Some caveats are in order. The proposed plots are tools that may facilitate the understanding of epidemic outbreaks or communication about outbreaks, but their utility is not established at this point. Logarithm transformations of data may cause interpretation difficulties for some readers, including scientists [9]; we would expect that many policy makers or members of the general public would appreciate that the proposed graphs be accompanied by a commentary and explanation (such as the video posted on Covid Trends website [7]). We have not conducted any tests of usability or impact.

Furthermore we suggest that the proposed graphs be acompanied by classic epidemic plots, as each graph highlights different features of an outbreak. Any graphical display entails a choice regarding what features of a dataset should be shown; no graphical method is neutral in this regard. Only experience will tell if these plots prove useful.

The similarity of visual patterns between real-life plots and the SIR model does not imply that the model is an exact representation of COVID-19 epidemics. The slow-down of a SIR epidemic is attributable to the depletion of susceptible individuals and the emergence of herd immunity. Seroprevalence data on SARS-CoV-2 (the virus which causes COVID-19) suggest that this is an unlikely explanation [10, 11]. The control of COVID-19 epidemics in most countries must be mainly due to other factors, such as measures taken to reduce the frequency of transmissions [12, 13].

The available data on COVID-19 deaths are of variable quality. Some country reports exhibit a variability that is larger than expected, including low frequencies of COVID-19 deaths reported over the weekend, other country reports display a lower variability than expected from a random process, yet other countries have introduced post-hoc corrections that do not reflect the day-to-day accrual of events. These issues affect the representativeness of any epidemic curves, including the proposed plots.

Finally, whether these plots may help in forecasting epidemic trajectories is unclear. In a previous analysis that used the plot of log(N) as a function of log(C) applied to cases of COVID-19 in Italy in March 2020, the power law predicted correctly the number of new cases recorded during the following week, before the lockdown effect became visible [3]. To what extent this method can be successfully generalized is currently not established.

## Conclusions

The plot of the logarithms of new deaths (or cases) N(t) as a function of cumulative deaths (or cases) C(t) allows simultaneous consideration of growth and size of an epidemic outbreak; it also allows the identification of exponential, sub-exponential, and linear growth. Furthermore, this graph facilitates comparisons of outbreaks of various sizes and the monitoring of outbreaks over time. The plot of the logarithm of the relative growth rate over time displays a key parameter of epidemic outbreaks, and helps identify deviations from a pattern of linear decrease that characterizes exponential epidemic decay.

## Supplementary information

**Additional file 1.**

## Data Availability

The dataset analysed during the current study is available from the European Centre for Disease Prevention and Control. COVID-19. Today’s data on the geographic distribution of COVID-19 cases worldwide. https://www.ecdc.europa.eu/en/publications-data/download-todays-data-geographic-distribution-covid-19-cases-worldwide

## Abbrevations

SIR: Susceptible-infected-removed model
