# Correction to: Alternative graphical displays for the monitoring of epidemic outbreaks, with application to COVID-19 mortality

**DOI:** 10.1186/s12874-020-01147-z

**Published:** 2020-10-26

**Authors:** Thomas Perneger, Antoine Kevorkian, Thierry Grenet, Hubert Gallée, Angèle Gayet-Ageron

**Affiliations:** 1 Division of clinical epidemiology, Geneva University Hospitals, and Faculty of medicine, University of Geneva, Geneva, Switzerland; 2 Teem Photonics, 61 Chemin du Vieux Chêne, 38240 Meylan, France; 3 Neel Institute, Université Grenoble Alpes, Grenoble, France; 4 Institute of Environmental Geosciences, Université Grenoble Alpes, Grenoble, France

**Correction to: BMC Med Res Methodol 20, 248 (2020)**

**https://doi.org/10.1186/s12874-020-01122-8**

Following the publication of the original article [1], it was noted that due to a typesetting error the Fig. 4 and Fig. 5 have been switched, the figure captions are correct.

**Fig. 4:**
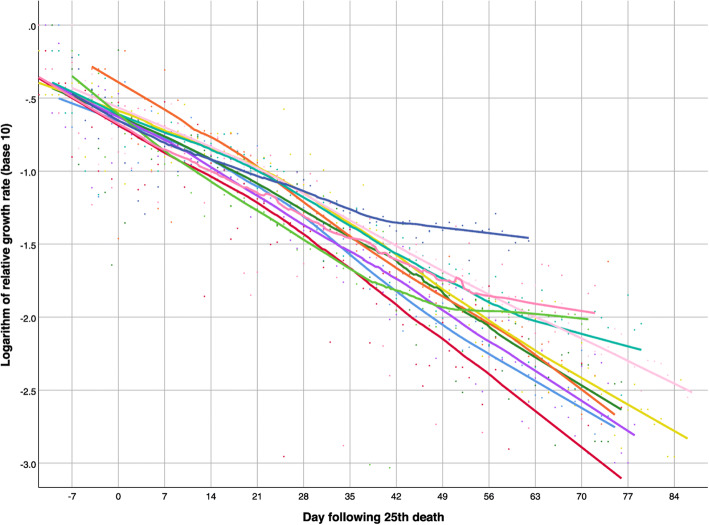
Logarithm of relative growth rate of deaths attributed to COVID-19 over time in 11 European countries, as of June 5, 2020 (from top right down): Russia (navy blue), Italy (light pink), Sweden (dark pink), Portugal (light green), United Kingdom (teal), France (mustard), Spain (orange), Germany (dark green), Netherlands (purple), Belgium (light blue), Switzerland (red). Smoothed lines were obtained by non-parametric regression

**Fig. 5:**
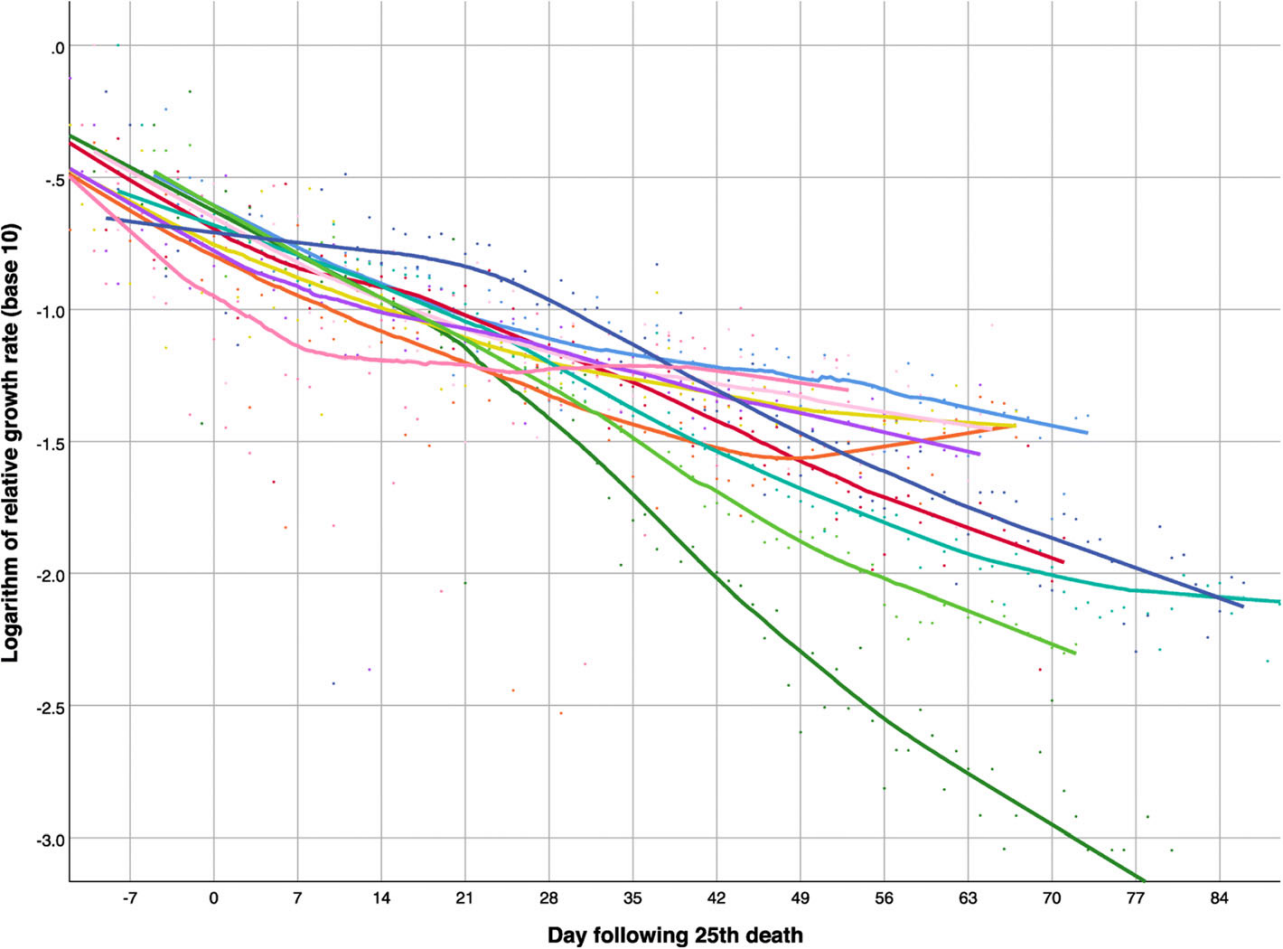
Logarithm of relative growth rate of deaths attributed to COVID-19 over time in 11 non-European countries, as of June 5, 2020 (from top right down): Brazil (light blue), South Africa (dark pink), Mexico (light pink), India (mustard), Peru (purple), Egypt (orange), USA (navy blue), Canada (red), Turkey (light green), Iran (teal), China (dark green). Smoothed lines were obtained by non-parametric regression

The correct figures and captions have been included in this correction, and the original article has been corrected.

## References

[1] Perneger (2020). Alternative graphical displays for the monitoring of epidemic outbreaks, with application to COVID-19 mortality. BMC Med Res Methodol.

